# Hybrid Machine Learning Technique for Forecasting Dhaka Stock Market Timing Decisions

**DOI:** 10.1155/2014/318524

**Published:** 2014-02-19

**Authors:** Shipra Banik, A. F. M. Khodadad Khan, Mohammad Anwer

**Affiliations:** School of Engineering and Computer Science, Independent University, Dhaka 1229, Bangladesh

## Abstract

Forecasting stock market has been a difficult job for applied researchers owing to nature of facts which is very noisy and time varying. However, this hypothesis has been featured by several empirical experiential studies and a number of researchers have efficiently applied machine learning techniques to forecast stock market. This paper studied stock prediction for the use of investors. It is always true that investors typically obtain loss because of uncertain investment purposes and unsighted assets. This paper proposes a rough set model, a neural network model, and a hybrid neural network and rough set model to find optimal buy and sell of a share on Dhaka stock exchange. Investigational findings demonstrate that our proposed hybrid model has higher precision than the single rough set model and the neural network model. We believe this paper findings will help stock investors to decide about optimal buy and/or sell time on Dhaka stock exchange.

## 1. Introduction 

People have a tendency to spend in stock market because of its higher returns over time. In the most important financial markets around the world, trading in share market has achieved remarkable recognition to obtain massive earnings. As a result, any awareness of potential information concerning price performance of a particular share will entirely guarantee huge profits in this market. Thus, proper forecast of this market is an important factor for investors, buyers, sellers, fund managers, policy makers, researchers, applied workers, and many others who are engaged in this market. However, in practice, share market prediction has been a complicated task because this market is extremely exaggerated by many interconnected political, economic, and even psychological reasons. These reasons interrelate with each other in a composite way; hence, it is hard to predict movements of a share market.

However, investigation and forecasting in share market has been a hot lesson for many years (e.g., [1–5] and others). Usually, in a stock market, techniques employed to formulate investment choices fall into two broad categories: (a) fundamental analysis and (b) technical analysis. Fundamental analysis is a complete method that involved real and reliable information of a company's financial report, economic conditions, and competitive strength. This technique believes that present price depends on its fundamental value, expected return on investment, and new information about a corporation that will affect movement of its share cost. Alternatively, technical analysis simply believes real record of trading and cost in a stock. It is identified that in predicting market progress, about 90% of stock traders use this technique in their investment study. This is mainly psychological analysis of market contributors and typically concerned with market indicators, which look at the trend of price indices and individual stocks. The prime theory of these indicators is that once a trend is in motion, it will persist in that track. Relative strength index, moving average, Bollinger bands, moving average convergence/divergence, price rate of change, and others have been commonly used technical indicators used to examine the trend of a market track via diagram presentations. To make huge profit from the share market, progressively most excellent forecasting methods are used by several analyzers. Currently, analyzers rely on multiple methods to get information about the future markets. This paper explains development about the share market prediction using data mining techniques. In recent years ([1, 6] and others), many researches in stock market forecasting are performed using computational intelligence methods and have shown higher prediction results. Such computational intelligence methods involve artificial neural network (ANN), rough set (RS) theory, fuzzy logic, genetic algorithm, bee colony method, ant colony method, and others.

The prime focus of this paper is to forecast Dhaka share market movements using the most popular and recently used RS and ANN forecasting models. The problem studied here is about the stock prediction for investors' usage. We have chosen ANN model of superior ability of knowledge discovery and RS model for powerful rules extraction abilities. We wish to extract knowledge in theform of rules from the daily Dhaka stock movements that would guide investors, buyers, sellers, and others whether to buy, sell, or hold a share. The most important technical indicators are used to create RS model and ANN model. The paper is planned as follows.  Section 2 talks about suggested prediction models. A short explanation about technical indicators is given in Section 3. Experimentation is enclosed in Section 4 including data preparation, analysis, results, and discussion of results. Finally, some concluding remarks and future works are provided in Section 5.

## 2. Proposed Prediction Models 

The RS model and the ANN model have been employed in share market prediction [1, 3, 4]. Based on earlier researches, both models have revealed capability in this application. The RS model has revealed successful results with superior precision [4, 6, 7]. Many RS models have been developed for several areas including many applications: analysis of share market data [1–5], forecasting [8], feature selection [9, 10], financial and investment areas [3], and many others. A detailed analysis of applications of RST in financial field can be found in [6]. Like RS, the ANN model has been used to forecast stock market for the past few years [11, 12] and is still being investigated by many researchers with the goal of achieving higher and perfect prediction. Based on successful results in applied literature given by RS and ANN in stock market prediction, we have chosen the following models to predict Dhaka share market. These are models based on (a) ANN, (b) RS, and (c) hybrid model of ANN and RS (ANN_RS).

### 2.1. ANN Prediction Model

**Figure 1:**
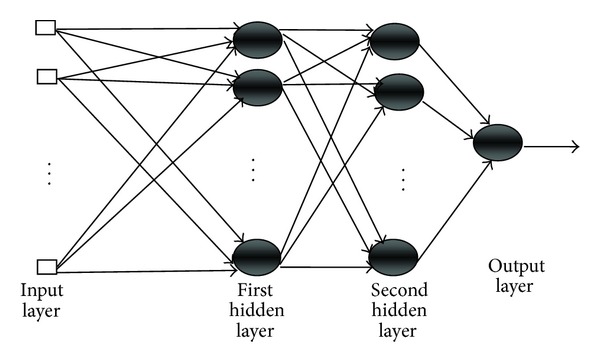
An ANN network.

It is a model (introduced by McCulloch and Pitts [13]) developed for simulating biological nervous systems such as the human brain. It has the following processing functions: receiving inputs, assigning appropriate weight coefficient of inputs, calculating weighted sum of inputs, comparing this sum with some threshold, and finally determining an appropriate output value.  Figure 1 presents a basic structure of ANN, which has 1 input layer, two hidden layers (with sufficient no. of neurons), and 1 output layer. Thus, each neuron receives an input *P*_1_, which is multiplied by weights *W*_*R*_ and bias *b*_1_ to produce the net input as *n* = *WP* + *b*, where *R* represents number of elements in input vector and *N* represents number of neurons in hidden layers. Passing net input through an activation function produces output of neurons. Usually, sigmoid function [*y* = *f*(*x*) = 1/(1 + *e* ^−*x*^)] is used as the activation function. The properties of this function need to mimic nerve cell which either fires or does not fire. Other used activation functions are hard limiter, pureline, transig, logsigmoid, and others. Networks are trained so that a particular input leads to a specific target output. The training algorithm is the standard black propagation (BP), which uses gradient descent (GD) technique to minimize error over all training data. During training, each desired output is compared with actual output and calculates error at output layer. The backward pass is error BP and adjustments of weights. Thus, the network is adjusted based on a comparison of output and target until network output matches target. After training process is completed, network with specified weights can be used for testing a set of data different than those for training. For details, see [7].

### 2.2. RS Prediction Model (RSPM)

It is developed (introduced by Pawlak [14]) based on mathematical tool to deal with vagueness and uncertainty in classification of objects in a set. In RS, data is organized in a table called decision table, containing attributes as columns and data elements as rows. The class label is called decision attribute. The rest of attributes are condition attributes. For rows, RST employs notion of indiscernible class, while for columns it employs notion of indiscernible attribute to identify significant attributes. The key idea of this approach lies in the analysis of limits of discernibility. RST defines three regions based on equivalent classes induced by attribute values: lower approximation, upper approximation, and boundary. The lower approximation is concerned with all objects which definitely belong to the set. The upper approximation consists of all objects which probably belong to the set. The boundary is the difference between upper approximation and lower approximation. Based on concept of indiscernibility relation, redundant features can be identified and eliminated to reduce number of features. Thus, RST is suitable for data reduction and very useful as a preprocessing tool. The advantage of rough set is that it does not need any preliminary information about data, for example, probability distribution of data and grade of membership like fuzzy set theory (details see [14]). Analysis of data by RS can be divided into five steps: constructing information table, identifying indiscernibility relations, finding reducts, and generating rules and finally classification. An information table is in the form of rows and columns that represent original data. The set of indiscernibility relations based on information table are derived using objects with set of features. The upper and lower approximations are used to deal with inconsistent objects that probably belong to the set. The main concern of RST is to find the smallest subset (known as reducts, computed by discernibility matrix) of features without losing any information. Reducts are sets that contain same quality of sorting whole original set of features but possess least features. From reducts, production rules to classify objects are generated by logical statements of type IF-THEN condition. The decision rules are measured by support, length, coverage, and accuracy. The rule support is number of records that fully exhibit the property described by IF-THEN condition. The length is defined as number of conditional elements of IF part. The coverage is defined as proportion of records that are identified by IF or THEN parts. The accuracy measures reliability of rule in THEN parts. If coverage is 1 for a rule, then this rule is known as complete; it means that any objects belong to class while deterministic rules are rules with accuracy equal to 1. The rules are correct with both coverage and accuracy equal to 1. For a detailed report of RS, see [14].

## 3. Technical Indicators 

The following most widely used indicators were used in this study: moving average over a 5-day period (MA5), moving average over a 12-day period (MA12), price rate of change (PROC), relative strength index (RSI), and moving average convergence/divergence (MACD). A very brief description about above considered indicators with interpretation is described as follows.

*(A) MA5.* By MA, a trader is able to understand the strength of the long-term trend of the prices. MA5 is the 5-day moving average. It is calculated by adding the last 5 indexes together and then dividing by 5.

*(B) MA12*. MA12 is the 12-day moving average. It is formulated by adding the last 12 indexes together and then dividing by 12.

*(C) PROC*. PROC attribute is a price momentum indicator. It is calculated by the following formula:

(1) $$\begin{array}{c} \frac{\left(\text{today's index}-\text{index}\;n\;\text{periods}\;\text{ago}\right)}{\text{index}\;n\;\text{periods}\;\text{ago}}. \end{array}$$

If the stock's price is higher (lower) today than *n* periods ago, PROC will be a positive (negative) number. As the security's price increases (decreases), its PROC will rise (fall). Faster prices rise (or fall) and faster PROC will rise (or fall). Thus, PROC values indicate an overall picture of trend strength generation.

*(D) RSI*. One of the most popular technical analysis indicators, RSI (developed by Wilder [15]) is an oscillator that measures current price strength in relation to previous prices. It is calculated as ratio of two exponentially smoothed MA. Mathematically, it is defined as

(2) $$\begin{array}{c} \text{RSI}=100-\left(100\left(1+R\right)\right),\;0<\text{RSI}<100, \end{array}$$

where *R* = AG/*AL*⁡, AG is average price gain over some periods, and AL is average price drop over some periods. RSI indicates internal strength of price. It is used to generate buy and sell signals. It also shows overbought and oversold conditions that confirm price movement and warn of potential price reversals through divergences. If we choose (for example) two references lines at 30 and 70 and if we observe RSI dips below 30 lines, a buy signal is generated. Likewise, if RSI exceeds 70 lines, a sell signal is generated. 

*(E) MACD*. It is an oscillator function used by technical analysts to spot overbought and oversold conditions. MACD is calculated by subtracting values of a 26-period exponential MA from a 12-period exponential MA. As its name implies, MACD is all about convergence and divergence of two MAs. Convergence occurs when MAs move towards each other. Divergence occurs when MAs move away from each other. The shorter MA (12 days) is faster and responsible for most MACD movements. The longer MA (26 days) is slower and less reactive to price changes in underlying stock.

The above technical indicators are used as dependent attributes in our analysis. The decision attribute is the trend of stock market and can be used to make decisions.

## 4. Data, Experimentation, and Results

**Figure 2:**
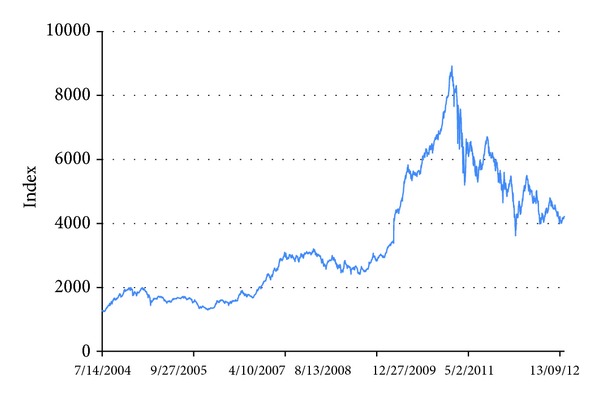
Time plots of the stock index.

To assess and validate prediction ability of the RS model, the ANN model, and the hybrid ANN_RS model, daily stock movement of all stocks traded in Dhaka stock exchange (DSE) and spanning over a period of 8 years (Jan 2004–December 2012) were captured (data source: http://www.dse.com.bd).  Table 1 represents a sample of the stock's daily movements and Figure 2 shows stock's movements w.r.t. time. We can observe that there has been an increasing trend of the prices up to April 2010. Then, there is a (seems to be) collapse in market observed after that. Certainly, there are some reasons for those changes which could be political, economic, and/or psychological. For details, see http://www.dse.com.bd.

Numerical statistical properties of the selected stock index are examined first before applying it to chosen forecasting models and reported in Table 2. We have tabulated selected attributes (MA5, MA12, PROC, RSI, and MACD) used in the creation of RS decision table and inputs to the ANN model in Table 3. These attributes are calculated from the DSE general index. The decision attribute *D* in this table indicates the future direction of data set and is made using the following rule:

(3) $$\begin{array}{c} D=\frac{\sum_{i=1}^{n}\left(\left(n+1\right)-i\right)\mathrm{sign}\left\lceil \text{index}\left(i\right)-\text{index}\left(0\right)\right\rceil }{\sum_{i=1}^{n}i}, \end{array}$$

where index(0) is today's index and index(*i*) is *i*th index in future. The above equation specifies a range −1 to +1 for *D*. A value of −1 indicates that next day's price is lower than that of current date, 0 indicates no change, and +1 indicates that next day's price is higher than that of current date. From raw data, we executed data preparation tasks that resulted in a new information table with conditional attributes *A* = (MA5, MA12, PROC, RSI, and MACD) and a decision attribute *D*.

In the next section, we will create RS model, ANN model, and hybrid model of ANN and RS (ANN_RS) based on selected technical indicators.

### 4.1. RS and ANN Model Building

The ANN_RS hybridizes high generality of ANN and rules extraction ability of RST. Data are divided into 2 parts: training and testing sets. The training set contains 70% of the collected data and testing set contains remaining data.

#### 4.1.1. Evaluation Methods

Confusion matrix is applied to assess performance of observed and predicted classes for selected models. This matrix is a table summarizing the number of true positive (TP) class, false positive (FP) class, false negative (FN) class, and true negative (TN) class. For example, TP means that output of prediction model rises and also that stock price actually rises and so on.

#### 4.1.2. Prediction Model—RS Model

The process of stock market data prediction and analysis is illustrated in the following steps: (i) create efficient indicators based on data, (ii) select training data set and test data set, (iii) place into RS model, (iv) extract trading rules, and (v) apply in real market. The RS analysis of data involved calculation of reducts from data, derivation of rules from reducts, rule evaluation, and prediction processes. The Rosetta Rough Set Toolkit [16] was used to perform reducts and create decision rules. The reducts that were produced from our selected data are shown in Table 4. We used Johnson's reducer algorithm and the equal binning discretized method.  Table 5 shows a partial set of generated rules. These obtained rules are used to make prediction systems. From our chosen data, we got a set of 12 reducts. The following is an example of a rule obtained from reduct 1 in Table 4.

The rulein Algorithm 1 has 3 conditional attributes corresponding to IF part. The rule has a decision of −1 or 1. From this rule we can see that conditional attributes have a support of 194 objects from a total of 952 objects. Of those 194 objects, 122 objects (63%) have a decision value of −1 or 1. We are looking for rules with relatively high support and high precision. Once rules were obtained, testing of each rule ensured that knowledge was accurate. Each rule fired against testing set to verify support, precision, and confidence measures. Comparison between measures was obtained by firing rules against training and testing data is needed to make sure that knowledge is a correct illustration of raw data.

The confusion matrix for the RS model is provided in Table 5.

On average, RS model provides 71.52% prediction accuracy of falling stock prices, 80.34% prediction accuracy of rising stock prices, and overall 87% prediction accuracy. Therefore, we can say that this model is 72% useful to predict falling stock and 87% useful to predict rising stock.

#### 4.1.3. Prediction Model—ANN Model

An ANN topology of 9 : 15 : 18 : 1, learning rate 0.01, and momentum factor parameter 0.90 is chosen using error and trial method. We used sigmoid transfer function at input and output layers and trained network by Levenberg Marquardt back-propagation algorithm. The learning rate parameter controls step size in each iteration and momentum parameter avoids getting stuck. Inputs to ANN model are index_*t*_; index_*t*−*i*_ (*i* = 1,2, 3,…*p*, *p* is AR order) MA5, MA12, PROC, RSI, and MACD; and the output index_*t*+1_. We have selected lag order using Akaike Information Criteria (AIC). Other criteria such as BIC, SIC, and others can also be used. The confusion matrix is provided in Table 6. On average, ANN model provides 73.43% prediction accuracy of falling stock prices, 81.63% prediction accuracy of rising stock prices, and overall 78.88% prediction accuracy. It is remarkable that although this model performs better than RS model to predict falling stock prices, it performs poorly for overall prediction. The reason rate of prediction for the class of FN is higher compared to the RS model.

#### 4.1.4. Model Combinations—The ANN_RS Model

Under 4.1.2 and 4.1.3, RS and ANN models are constructed alone as baselines; then, these two models are combined to improve rate of prediction precision. First, an ANN topology of 9 : 12 : 14 : 1, learning rate 0.01, and momentum factor parameter 0.95 is selected by error and trial technique. Then, Rosetta Rough Set Toolkit was used to execute reducts and create decision rules based on predicted index. To observe performance of model, confusion matrix is reported in Table 7. After developing two different baseline models, we see that RS model provides better performances than ANN model. The hybrid model ANN_RS provides the 95.29% prediction accuracy of falling stock prices, 96.33% prediction accuracy of rising stock prices, and overall 97.68% prediction accuracy.

From evaluation among models RS, ANN, and hybrid ANN_RS, we can realize that the hybrid ANN_RS model has better forecasting performances than others. That means that this model has superior average prediction precision. Therefore, according to our study, the hybrid model can be recommended to predict the daily Dhaka stock movements that would guide buyers, sellers, investors, and others when to buy, sell, or hold a share.

## 5. Concluding Remarks

To decide optimal buy and sell time on DSE, a design of the hybrid machine learning model is presented in this paper. The ANN forecasting model and the RS forecasting model are combined to improve rate of prediction precision and to provide decision rules whether to buy, sell, or hold a stock. Results of this proposed hybrid model are compared for the baseline RS model and the ANN model. To enhance efficiency of prediction procedure, the RS equal binning discretized method is used to discretize the data. Then, the RS Johnson's reducer algorithm is applied to find all reducts of data containing minimal subset of attributes. Confusion matrix is used to assess performance of chosen models and classes (fall and rise). The experimental result shows that our proposed hybrid model has 97% accuracy which is higher than the single RS forecasting model and the ANN forecasting model. Other forecasting models, for example, ANFIS, genetic algorithm, can be applied for further evaluations. To gain better prediction, other superior reduction techniques such as Holte's algorithm, genetic algorithm, can be also applied. These are left for future works.

## Figures and Tables

**Algorithm 1:**
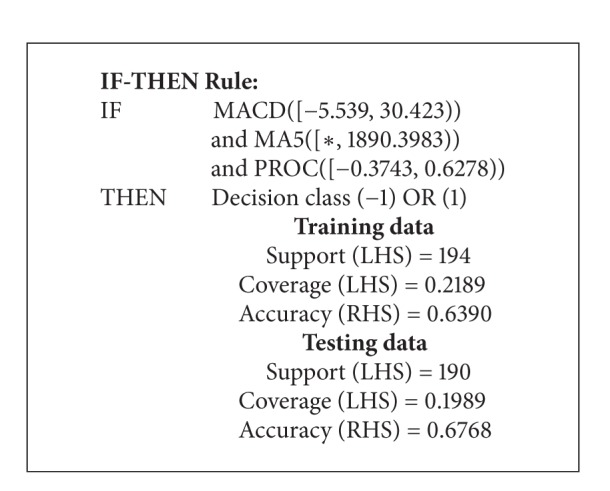


**Table 1:** Daily Dhaka stock price movement.

Date	Total trade	Total volume	Total value in Taka (mn)	Total market cap. in Taka (mn)	DSE general index
06/07/12	79537	56802386	2989.670	2551077.1	4769.394
06/10/12	36858	27487186	1363.139	2532069.0	4725.725
06/11/12	44550	34578322	1564.310	2517980.5	4689.201
06/12/12	41760	29559249	1287.442	2547020.2	4691.297
06/13/12	51959	36631286	1496.727	2516529.1	4680.616

**Table 2:** Numerical summaries of stock price.

Minimum	Maximum	Mean	SD	Skewness	Kurtosis
1.199*e* + 003	9.901*e* + 003	3.378*e* + 003	1.9197*e* + 003	0.8723	2.6224

**Table 3:** Sample of Dhaka stock index after postprocessing.

Date	Total trade	Total volume	Total value in Tk (mn)	Total market cap. in Tk (mn)	DSE general index	MA5 (1000)	MA12 (1000)	PROC	RSI	MACD	*D*
6/3/2012	79024	52834504	2773.02	2582225.4	4855.36	4.71	4.82	−3.694	36.57	−97.68	−1
6/4/2012	98570	64218144	3279.275	2509830.4	4675.98	4.72	4.79	1.410	30.74	−101.5	1
6/5/2012	59303	38597358	1956.071	2537026.4	4741.94	4.74	4.77	1.055	35.86	−98.23	1
6/6/2012	72496	62699058	3079.895	2557495.7	4791.98	4.76	4.75	−0.471	39.82	−90.49	−1
6/7/2012	79537	56802386	2989.67	2551077.1	4769.39	4.77	4.74	−0.916	39.69	−85.19	−1
6/10/2012	36858	27487186	1363.139	2532069	4725.73	4.74	4.73	−0.773	34.80	−83.56	−1

**Table 4:** Generated reducts.

Reduct #	Reduct
1	{MACD, MA5, PROC}
2	{PROC}
3	{PROC, RSI}
4	{MACD, MA5, MA12, PROC}
5	{MA12, PROC, RSI}
6	{MA5, MA12, PROC}
7	{MACD, PROC, RSI}
8	{MA5, PROC, RSI}
9	{MACD, MA5, RSI}
10	{MA5, MA12}
11	{MACD, MA12, PROC, RSI}
12	{MACD, MA5, PROC, RSI}

**Table 5:** Confusion matrix for the RS model.

Actual	Projected		Accuracy (%)
	Fall (−1)	Rise (+1)	
Fall (−1)	314	125	0.71526195
Rise (+1)	2	511	1.0
Accuracy (%)	1.0	0.803459119	0.866596638

**Table 6:** Confusion matrix for the ANN model.

Actual	Projected		Accuracy (%)
	Fall (−1)	Rise (+1)	
Fall (−1)	293	106	0.7343358396
Rise (+1)	95	458	0.8282097649
Accuracy (%)	0.755154	0.816399	0.7888655462

**Table 7:** Confusion matrix for the ANN_RS model.

Actual	Projected		Accuracy (%)
	Fall (−1)	Rise (+1)	
Fall (−1)	405	20	0.9529411765
Rise (+1)	2	525	0.9962049336
Accuracy (%)	0.99508599	0.963302752	0.9768907563
